# The analysis of lysine succinylation modification reveals the mechanism of oxybenzone damaging of pakchoi (*Brassica rapa* L. ssp. *chinensis*)

**DOI:** 10.3389/fpls.2022.1001935

**Published:** 2022-12-08

**Authors:** Shuhao Li, Yuqi Zhou, Yang Xu, Shengxiang Ran, Maomao Hou, Qingming Li, Xin Zhong, Fenglin Zhong

**Affiliations:** ^1^ College of Horticulture, Fujian Agriculture and Forestry University, Fu’zhou, China; ^2^ Institute of Urban Agriculture, Chinese Academy of Agricultural Sciences, Cheng’du, China; ^3^ Institute of Marine Science and Technology, Shandong University, Qing’dao, China

**Keywords:** oxybenzone, pakchoi, lysine succinylation, oxidative stress, EMP-TCA

## Abstract

Oxybenzone (OBZ), one of a broad spectrum of ultraviolet (UV) absorbents, has been proven to be harmful to both plants and animals, while omics analysis of big data at the molecular level is still lacking. Lysine succinylation (Ksuc) is an important posttranslational modification of proteins that plays a crucial role in regulating the metabolic network in organisms under stress. Here, we report the changes in intracellular Ksuc modification in plants under OBZ stress. A total of 1276 succinylated sites on 507 proteins were identified. Among these sites, 181 modified proteins were hypersulfinylated/succinylated in OBZ-stressed pakchoi leaves. Differentially succinylated proteins (DSPs) are distributed mainly in the chloroplast, cytoplasm, and mitochondria and are distributed mainly in primary metabolic pathways, such as reactive oxygen species (ROS) scavenging, stress resistance, energy generation and transfer, photosynthetic carbon fixation, glycolysis, and the tricarboxylic acid (TCA) cycle. Comprehensive analysis shows that Ksuc mainly changes the carbon flow distribution, enhances the activity of the antioxidant system, affects the biosynthesis of amino acids, and increases the modification of histones. The results of this study first showed the profiling of the Kusc map under OBZ treatment and proposed the adaptive mechanism of pakchoi in response to pollutants and other abiotic stresses at the posttranslational level, which revealed the importance of Ksuc in the regulation of various life activities and provides a reference dataset for future research on molecular function.

## Highlights

Ksuc plays an important role in the response of plants to oxybenzone (OBZ) stress.

Succinylated proteins are abundant in photosynthesis and carbon metabolism.

Ksuc regulates reactive oxygen species (ROS) resistance, C flow distribution, and energy production pathways.

## Introduction

Oxybenzone (OBZ), also known as benzophenone-3 (BP-3), is a kind of ultraviolet (UV) filter. OBZ is usually added to various products, as well as personal care products, pesticides, plastics, etc. (Jeon et al., 2006; Tsui et al., 2017), which can easily enter the aquatic and soil environment through human activities (for example, bathing, swimming, sewage discharge) (Meng et al., 2020). The concentration existing in the environment is between ng·L^−1^ and mg·L^−1^ (Downs et al., 2021). Most research has shown that OBZ can cause severe damage to ecosystems, including plants and animals. Wnuk et al. (2018) have shown that prenatal exposure to BP-3 induces apoptosis, disrupts oestrogen receptor expression, and alters the epigenetic status of mouse neurons. Teoh et al. (2020) found that OBZ could significantly affect the protein content of green microalgae. An early study by our team showed that OBZ could cause severe oxidative stress in cucumbers (Zhong et al., 2019; Zhong et al., 2020). Meanwhile, the widespread use of sewage irrigation technology further exacerbated the accumulation of UV filters (including OBZ) in terrestrial ecosystems. Thus, the damage of OBZ to plants has proven to be more severe, especially in the pathway of photosynthesis (Zhong et al., 2020; Wang et al., 2022).

A timely and effective response to these abiotic stresses is a prerequisite for plant survival (He et al., 2016). The plant response to environmental stress is reflected in many aspects. Compared with transcription and translation, protein posttranslational modifications (PTMs) can significantly affect the protein localization, secondary structure stability, and enzyme activity of the modified protein (Rosen et al., 2004), which helps trigger plants to respond to stress more quickly. Thus, research on the function of PTMs has become a major concern among scientists. Among PTMs, lysine succinylation (Ksuc) was first discovered in *Escherichia coli* (Zhang et al., 2011) and has been proven to exist widely in bacteria (Pan et al., 2015), fungi (Zheng et al., 2016), animals (Weinert et al., 2013), plants (Zhang et al., 2017) and other organisms. Research has shown that Ksuc modification (100 Da) can cause a large change in protein quality and can induce more charge mutation (valence from +1 to −1) (Zhang et al., 2011; Yang and Gibson, 2019) than lysine acetylation and methylation modification (valence from +1 to 0 or no change), which means that Ksuc could induce more changes in protein properties and play a crucial role in regulating the structure and operation of functional proteins (Zhang et al., 2011). Moreover, Ksuc is considered a potential mechanism for coordinating energy metabolism and reactive oxygen species (ROS) metabolism under specific circumstances, such as stress (Huang et al., 2021). Several recent studies have revealed the mechanism of plant response and adaptation to periodic albinism (Xu et al., 2017), grafting (Yuan et al., 2019), phosphorus deficiency, and restoration (Wang et al., 2021) through Ksuc analysis. This new technology provides comprehensive information on the interaction of organisms with environmental factors.

Pakchoi (*Brassica rapa* L. ssp. *chinensis*) is a leafy vegetable widely cultivated in most Asian countries. Due to its short growth cycle and high multiple cropping index, pakchoi has usually been used as a good experimental model in various studies (Huang et al., 2022; Shen et al., 2022). In this research, we used pakchoi-type species “Jinpin hanchun” as the material. With the technology of label-free quantitative succinylation proteomics, we attempted to decode the molecular adaptation mechanism of the response of higher plants to OBZ stress. Two main questions were focused on, that is, whether Ksuc is involved in the regulation of the plant response to OBZ stress and which metabolic pathways are mainly regulated by Ksuc. Our study first showed the profiling of Ksuc of plants under OBZ stress, which is helpful to further understand the damage of OBZ to higher plants at the posttranslational level, especially on the stress response and primary metabolic processes. Furthermore, our results extended the knowledge boundary of the biological functions of Ksuc and provided a data reference for future studies on the changes in Ksuc in plants under other environmental stresses.

## Materials and methods

### Plant material and treatments

Pakchoi seeds (“Jinpin hanchun”) were choosed in this study. After socking at 25°C to accelerate germination, the seedlings of the same size were selected and transferred to 72-hole 1.5 mL centrifugal tube plastic containers, which contained 400 mL of a 1/4 dose of Hoagland nutrient solution. The cultivated environment was controlled as 25 ± 3°C, 180 ± 15 μmol·m^−2^·s^−1^ photosynthetic photon flux density (PPFD) for a 12:12 light cycle. After 3 days of preculture, seedlings were treated with different OBZ solutions. To investigate the mechanism of plants’ response to OBZ stress, we perform preliminary experiments using a series of OBZ concentration gradients, the concentration of 11.4 mg·L^−1^ was selected because of the significant phenotypic changes on pakchoi. OBZ solution was prepared by first dissolving OBZ (98%, Sigma-Aldrich, Burlington, MA, USA) in absolute ethanol and then placing it into 1/4 dose of Hoagland nutrition solution, the final alcohol content in the solution in both the control and OBZ groups was 0.1%. Then the seedlings were treated for 7 days for experiments. And the leaf tissue was immediately frozen in liquid nitrogen and then stored in a -80°C refrigerator for omics sequencing and analysis.

### Growth

The measurements of the shoot and root fresh weight were performed using an electronic balance (precision 0.0001 g, FB124, Shanghai Sunny Hengping Instrument Co., Ltd., Shanghai, China). The difference in fresh weight of the control and the OBZ treatment groups before and after 7 days was calculated respectively, as the changes in the fresh weight of the shoots and roots of each group.

### Protein extraction and trypsin digestion

The method of protein extraction and trypsin digestion was according to Liu et al. (2021) and Jin et al. (2021). Briefly, after being treated with OBZ for 7 days, the leaf tissues of the two groups were used for label-free succinylated proteomic analysis by PTM BioLab, Inc. (Hangzhou, China). Three replicates were set in each treatment. The sample was ground with liquid nitrogen, then transferred to a 5 mL centrifuge tube, and sonicated 3 times on ice using a high-intensity ultrasonic processor (Scientz, Ningbo, China) in 4 volumes of lysis buffer. After adding an equal volume of Tris-saturated phenol (pH 8.0), the mixture was further vortexed for 5 min. After centrifugation (4°C, 10 min, 5,500 g), the upper phenol phase was transferred to a new centrifuge tube. Proteins were precipitated by adding 5 volumes of 0.1 mol·L^−1^ ammonium acetate-saturated methanol overnight. The precipitate was washed with ice-cold methanol, followed by ice-cold acetone 3 times. The protein was redissolved in 8 mol·L^−1^ urea and the protein concentration was determined with a BCA kit (Beyotime Biotechnology Co., Ltd., Shanghai, China) according to the manufacturer’s instructions. After being reduced with dithiothreitol and alkylated with iodoacetamide in darkness, protein samples were then diluted with TEAB. Finally, a different ratio of trypsin was added for twice digestion.

### Affinity enrichment

The method of affinity enrichment was according to Wang et al. (2021). In short, tryptic peptides dissolved in IP buffer were incubated with pre-washed antibody beads (Lot number PTM-402, PTM Bio) at 4°C overnight with gentle shaking, and washed with IP buffer and H_2_O for 4 and 2 times respectively. Then, trifluoroacetic acid (0.1% concentration) was used for the bound peptides elution, and the eluted components were combined and vacuum-dried and desalted with C18 ZipTips (Millipore) according to the manufacturer’s instructions for LC-MS/MS analysis.

### LC-MS/MS analysis

The method of LC-MS/MS analysis was according to Liu et al. (2021). Briefly, After dissolved in solvent A, the tryptic peptides were directly loaded onto a home-made reversed-phase analytical column, then peptides were separated with a gradient from 9% to 25% solvent B at a constant flowrate of 500 nL·min^−1^ on an EASY-nLC 1200 UPLC system (ThermoFisher Scientific). And separated peptides were analyzed in Q Exactive™ HF-X (ThermoFisher Scientific) with a nano-electrospray ion source.

### Bioinformatic analysis of Ksuc

The detected proteins were identified and quantified by comparison with the Chinese cabbage (*Brassica rapa* ssp. *pekinensis*, “Chiifu-401-42”) genome database, Blast_Brapa_genome_v3_20211219.fasta (46250 sequences), by using MaxQuant software (v1.6.15.0, Max Planck Institute of Biochemistry, Germany). The differentially succinylated proteins (DSPs) were defined as the succinylated proteins with a fold change (FC) > 1.5 or FC < 1/1.5 and *P* value < 0.05. The Eukaryotic Orthologous Groups (KOG) functional classification statistics of DSPs were performed by comparing them with the KOG database. Prediction of subcellular localization was performed by WoLF PSORT software. Amino-acid sequence motifs of 10 positions upstream and downstream of the sites were analyzed using MoMo analysis based on the Motif-X algorithm (http://meme-suite.org/tools/momo) with *P* value < 0.000001. All the protein sequences in the database were treated as background controls. The minimum number of occurrences was set to 20. The option “Emulate original motif-x” was selected and other parameters were set to default. Through the Search Tool for the Retrieval of Interacting Genes/Proteins (STRING) (v.11.0) protein interaction network database, the differentially succinylated proteins were subjected to protein-protein interaction (PPI) network analysis according to the principle of confidence score > 0.7 (high confidence) and visualized by the R package “networkD3” tool.

## Results

### Significant inhibition of OBZ on plant growth

**Figure 1:**
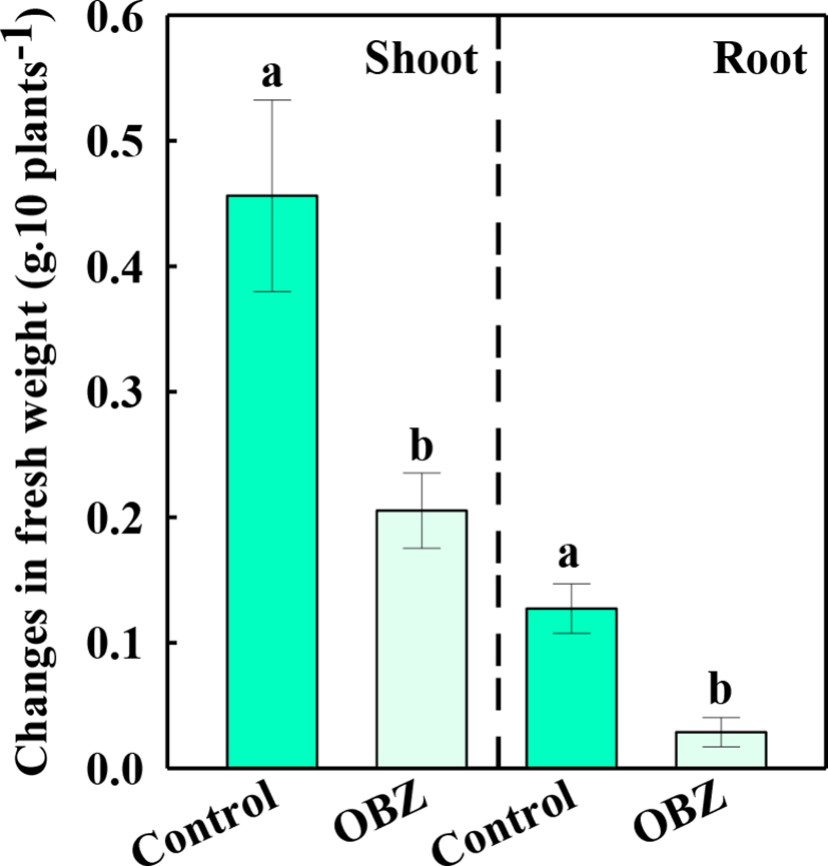
The changes in the fresh weight of the shoots and roots before and after being treated with 0 or 11.4 mg·L^−1^ OBZ for 7 days. Values are the mean ± SD (n = 6). Different letters indicate significant differences at *P* < 0.05 according to Tukey’s test.

After being treated with OBZ for 7 days, the increased fresh weight of the above and below ground of pakchoi was only 44.98% and 22.54% respectively compared to the increased content of the control group (**Figure 1**).

### Dynamic changes in the succinylated proteome in pakchoi leaves under OBZ treatment

**Figure 2:**
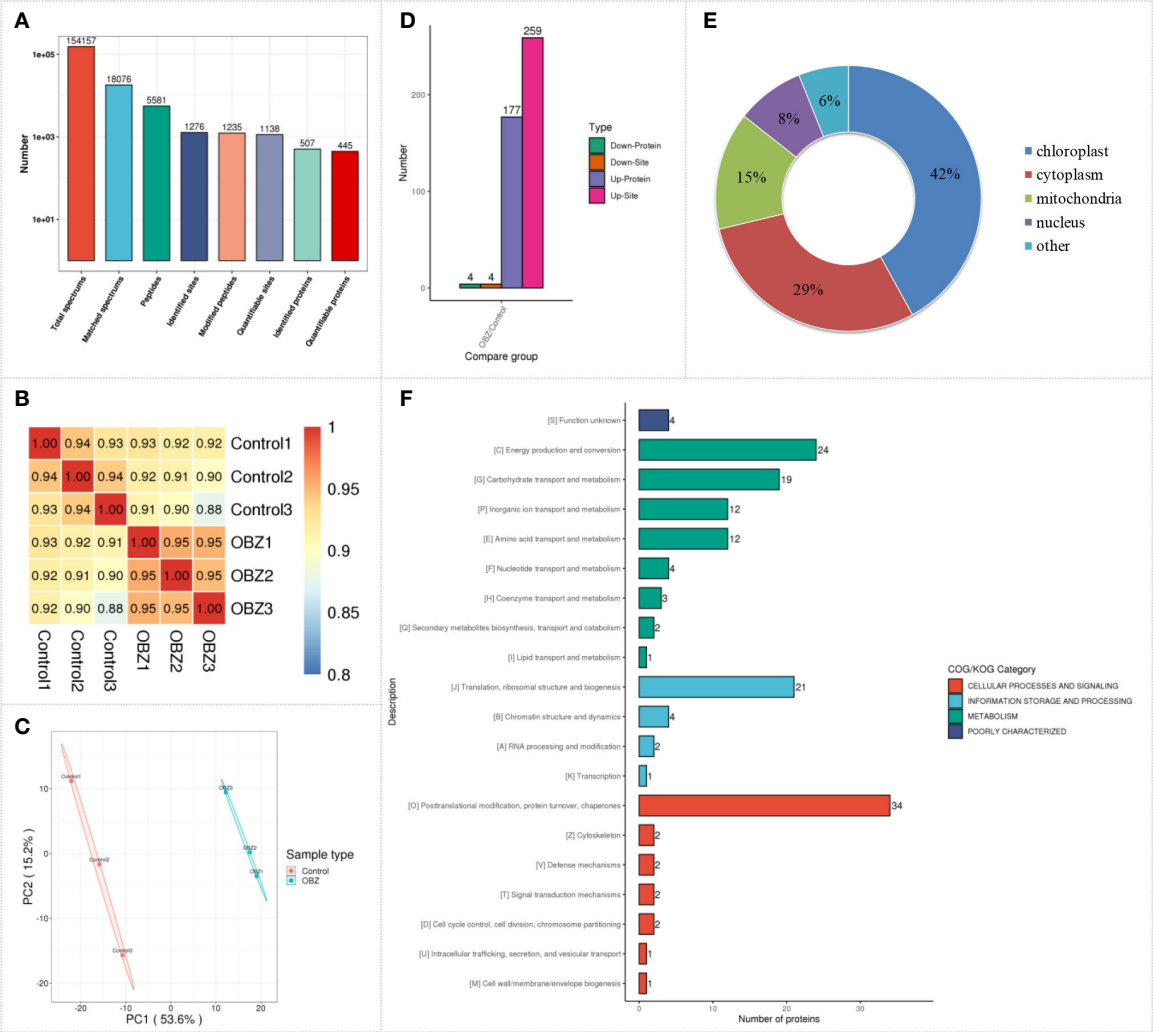
Characterization of lysine succinylation under OBZ treatment in pakchoi leaves. The number of identified proteins and sites containing the indicated lysine succinylation **(A)**, PCC analysis **(B)**, PCA analysis **(C)**, differentially expressed succinylation proteins and sites **(D)**, subcellular classify **(E)**, and KOG classifies **(F)**.

To quantify the succinylation modification level of pakchoi under OBZ treatment, we performed label-free quantitative succinylation proteomic analysis. After data preprocessing and deredundancy, a total of 1276 succinylated sites on 507 proteins were identified in this study (**Figure 2A** and **Supplementary Table 1**), and most of the identified proteins were succinylated at only one or two sites. Succinylation changes were strongly correlated across biological replicates, which showed excellent reproducibility, and the Pearson’s correlation coefficient (PCC) was greater than 0.93 (**Figure 2B**). Principal component analysis (PCA) showed that there were significant differences between the OBZ-treated and control groups in the level of succinylation (**Figure 2C**). Proteins with a fold change of more than 1.5 times and a significant difference between the control and the treatment groups (FC > 1.5 or FC < 1/1.5; *P* value < 0.05) were selected for subsequent analysis. Among these proteins, 263 differential succinylation sites of 183 proteins were identified under OBZ/control treatment, of which 259 sites of 177 proteins were upregulated, and 4 sites of 4 proteins were downregulated at the modification level (**Figure 2D** and **Supplementary Table 2**). Subcellular classification of these proteins was performed using bioinformatics, and they were found to be distributed mainly in the chloroplast, cytoplasm, mitochondria, and nucleus (**Figure 2E** and **Supplementary Table 2**). In addition, the results of functional classification analysis (KOG classification) showed that the different succinylated proteins were distributed mainly in the metabolic pathways of posttranslational modification, protein turnover, chaperones, energy production and conversion, translation, ribosomal structure and biogenesis, carbohydrate transport and metabolism, amino acid transport and metabolism, inorganic ion transport and metabolism, and chromatin structure and dynamics (**Figure 2F** and **Supplementary Table 2**). All the results suggest that plants could respond to abiotic stress by regulating the level of protein succinylation.

**Figure 3:**
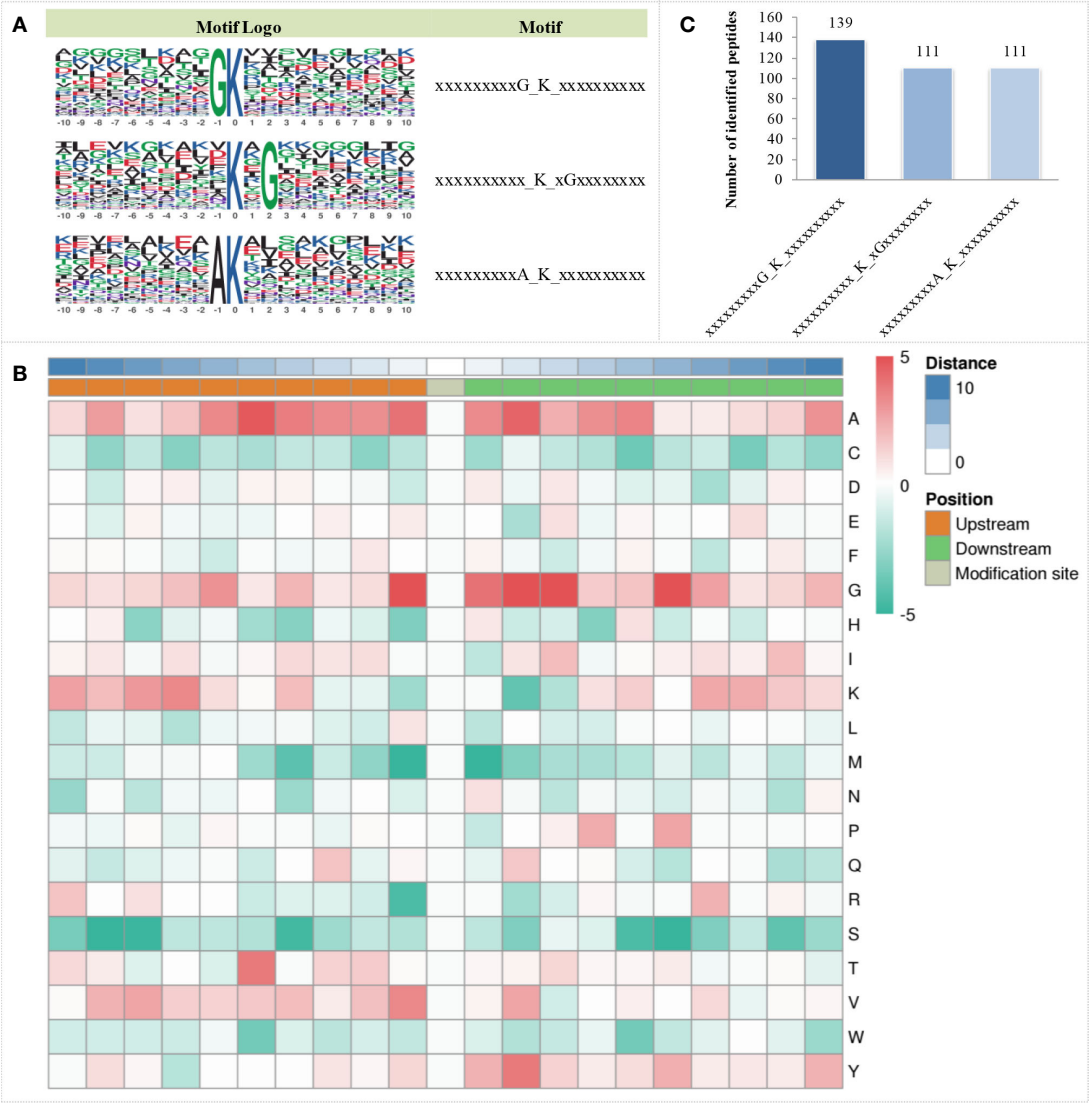
Sequence characteristics of succinylation proteins under OBZ treatment in pakchoi leaves. Analysis of the conserved motifs of succinylation proteins **(A)**, number of identified peptides containing the indicated succinylation motifs **(B)**, and characterization of amino acid sequences around the succinylation sites **(C)**.

The MoMo analysis tool based on the motif-x algorithm was used to analyse the motif characteristics of the modification sites. Three succinylation motifs were defined at 361 unique sites, which accounted for 28.29% of the total lysine succinylation sites (**Figure 3A**). These motifs showed different abundances, with the motif GK having the highest proportion of all identified peptides, while KG and AK had the lowest proportion of these motifs (**Figure 3B**). Based on these results, the analysis of the occurrence frequency of amino acids near lysine succinylation indicated that alanine (A) was at the positions of -1 to -6, 1 to 5, and 10; glycine (G) was at the positions of -1, -6, 1 to 3 and 6; lysine (K) was at the position of -7; threonine (T) was at the position of -5; valine (V) was at the position of -1; and tyrosine (Y) was at the position of 2. These positions are preferred in cells (**Figure 3C**).

### The antioxidant system response to OBZ stress

**Figure 4:**
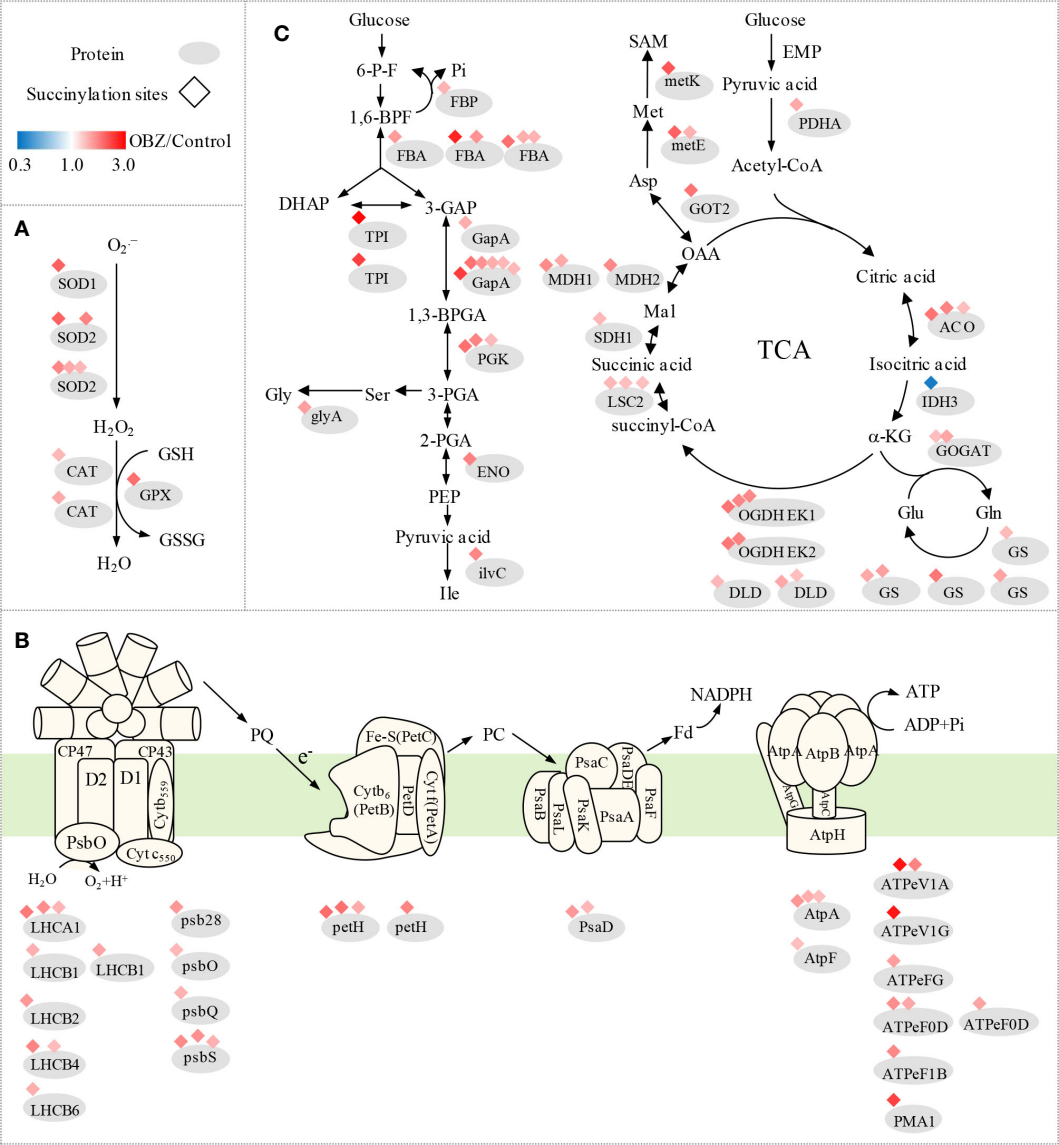
Succinylation of antioxidant enzymes **(A)** and central metabolic enzymes **(B, C)** in pakchoi leaves under OBZ stress.

Our results showed that OBZ treatment promoted the succinylation degree of antioxidants, including superoxide dismutase (SOD1, SOD2), catalase (CAT), glutathione peroxidase (GPX), and glutathione S-transferase (GST) (**Figure 4A** and **Table 1** and **Supplementary Table 3**). In addition, heat shock protein HSPA1S was also found to be hypersuccinylated.

**Table 1:** Differentially modified sites (modified proteins) for antioxidant system.

Protein accession	OBZ/Control Ratio	OBZ/Control *P* value	Regulated Type	KEGG Gene
BraA04g020160.3C	2.234	0.03833	Up	SOD1
BraA01g015820.3C	2.250	0.00320	UP	SOD2
BraA01g039910.3C	1.950	0.02620	Up	SOD2
BraA03g059190.3C	1.570	0.00560	UP	CAT
BraA07g015680.3C	1.670	0.01350	UP	CAT
BraA02g028350.3C	2.120	0.00210	UP	GPX
BraA03g016420.3C	3.490	0.00300	UP	GST
BraA03g024560.3C	2.700	0.00150	UP	GST
BraA03g024700.3C	1.800	0.00910	UP	GST
BraA07g026610.3C	4.770	0.00600	Up	GST
BraA03g051830.3C	1.900	0.00260	Up	HSPA1s
BraA10g033140.3C	2.410	0.01930	Up	HSPA1s

### Changes in photosynthesis and carbon metabolism

Our results showed that OBZ treatment promoted the degree of succinylation of light-harvesting complex (LHCA1, LHCB2, LHCB4, LHCB6), PSII subunit (psb28, psbO, psbQ, psbS, ferredoxin-NADP^+^ reductase (petH), PSI subunit (psaD), adenosine triphosphate (ATP) synthase subunit (AtpA, AtpF, ATPeV1A, ATPeV1G, ATPeFG, ATPeF0D, ATPeF1B), H^+^-transporting ATPase (PMA1), ubiquinol-cytochrome c reductase cytochrome c1 subunit (CYC1), fructose-1,6-bisphosphatase I (FBP), fructose-bisphosphate aldolase (FBA), triosephosphate isomerase (TPI), glyceraldehyde 3-phosphate dehydrogenase (GAPDH/gapA), phosphoglycerate kinase (PGK), enolase (ENO), pyruvate dehydrogenase E1 component alpha subunit (PDH E1K/PDHA), aldehyde dehydrogenase (NAD^+^) (ALDH), aconitate hydratase (ACO), 2-oxoglutarate dehydrogenase E1 component (OGDH E1k/OGDH), 2-oxoglutarate dehydrogenase E2 component (OGDH E2k/DLST), dihydrolipoamide dehydrogenase (DLD), succinyl-CoA synthetase beta subunit (LSC2), succinate dehydrogenase (ubiquinone) flavoprotein subunit (SDHA/SDH1) and malate dehydrogenase (MDH1, MDH2). In contrast, ribulose-bisphosphate carboxylase small chain (rbcS), glyceraldehyde 3-phosphate dehydrogenase (GAPA) and isocitrate dehydrogenase (NAD^+^) (IDH3) were significantly desuccinylation under OBZ treatment. (**Figures 4B, C**; **Table 2** and **Supplementary Table 4**).

**Table 2:** Differentially modified sites (modified proteins) for photosynthesis and carbon metabolism.

Protein accession	OBZ/Control Ratio	OBZ/Control *P* value	Regulated Type	KEGG Gene
BraA09g045720.3C	2.055	0.03290	Up	LHCA1
BraA05g010700.3C	1.625	0.00940	Up	LHCB1
BraA07g010780.3C	1.716	0.00180	Up	LHCB1
BraA09g003290.3C	1.812	0.00080	Up	LHCB2
BraA05g037360.3C	1.970	0.01530	Up	LHCB4
BraA06g011900.3C	1.631	0.02920	Up	LHCB6
BraA01g008860.3C	1.810	0.01220	Up	psb28
BraA01g022780.3C	1.568	0.00890	Up	psbO
BraA01g012030.3C	1.610	0.02710	Up	psbQ
BraA10g007760.3C	1.916	0.01180	Up	psbS
BraA07g016040.3C	2.153	0.00250	Up	petH
BraA09g009720.3C	1.972	0.02730	Up	petH
BraA09g001460.3C	1.809	0.00890	Up	psaD
BraA06g023070.3C	1.824	0.0390	Up	atpA
BraA01g005820.3C	1.506	0.02020	Up	atpF
BraA07g026480.3C	2.854	0.00944	Up	ATPeV1A
BraA05g040650.3C	2.825	0.00869	Up	ATPeV1G
BraA01g008130.3C	1.761	0.00646	Up	ATPeFG
BraA04g007070.3C	1.887	0.01971	Up	ATPeF0D
BraA09g043500.3C	1.715	0.03072	Up	ATPeF0D
BraA10g030970.3C	1.930	0.00568	Up	ATPeF1B
BraA09g011650.3C	2.481	0.00286	Up	PMA1
BraA09g003120.3C	2.537	0.00212	Up	CYC1
BraA09g044960.3C	1.595	0.01993	Up	FBP
BraA08g022720.3C	2.120	0.01829	Up	FBA
BraA01g017270.3C	1.777	0.01696	Up	FBA
BraA07g021000.3C	2.716	0.00014	Up	FBA
BraA04g004600.3C	2.933	0.01276	Up	TPI
BraA04g015400.3C	2.501	0.01637	Up	TPI
BraA06g009730.3C	2.529	0.00772	Up	gapA
BraA05g042370.3C	1.648	0.00676	Up	gapA
BraA07g042460.3C	2.121	0.00181	Up	PGK
BraA04g026020.3C	1.988	0.01382	Up	ENO
BraA09g039180.3C	1.682	0.00272	Up	PDHA
BraA06g018870.3C	1.683	0.02753	Up	ALDH
BraA08g026360.3C	1.683	0.02753	Up	ALDH
BraA03g042060.3C	2.105	0.01533	Up	ACO
BraA06g029260.3C	2.092	0.02011	Up	OGDH
BraA03g013590.3C	2.052	0.01651	Up	DLST
BraA06g004870.3C	1.684	0.01861	Up	DLD
BraA05g030240.3C	1.567	0.04078	Up	DLD
BraA09g013400.3C	1.561	0.00465	Up	LSC2
BraA07g017190.3C	1.558	0.03497	Up	SDHA
BraA10g002950.3C	1.917	0.00277	Up	MDH1
BraA08g001680.3C	1.926	0.01308	Up	MDH2

### Changes in the biosynthesis of amino acids

Our results showed that OBZ treatment significantly upregulated the succinylation level of glycine hydroxymethyl transferase (glyA/SHMT), ketol-acid reductoisomerase (ilvC), glutamine synthetase (GS), glutamate synthase (GOGAT), aspartate aminotransferase (GOT2), 5-methyltetrahydropteroyltriglutamate-homocysteine methyltransferase (metE), S-adenosylmethionine synthetase (metK) and 4-aminobutyrate-pyruvate transaminase (POP2). On the contrary, glutamate-glyoxylate aminotransferase (GGAT) was desuccinylation under OBZ treatment (**Figure 4C**; **Table 3** and **Supplementary Table 5**).

**Table 3:** Differentially modified sites (modified proteins) for biosynthesis of amino acids.

Protein accession	OBZ/Control Ratio	OBZ/Control *P* value	Regulated Type	KEGG Gene
BraA01g001110.3C	1.757	0.02059	Up	glyA
BraA07g023500.3C	1.949	0.03692	Up	ilvC
BraA03g038160.3C	2.079	0.03048	Up	GS
BraA02g006550.3C	1.751	0.00126	Up	GS
BraA04g010460.3C	1.726	0.00006	Up	GS
BraA08g009150.3C	1.546	0.01846	Up	GS
BraA10g032170.3C	1.696	0.04482	Up	GOGAT
BraA04g022190.3C	2.09	0.01089	Up	GOT2
BraA10g022160.3C	2.232	0.00706	Up	metE
BraA03g037900.3C	2.323	0.00272	Up	metK
BraA01g031790.3C	3.322	0.00385	Up	POP2
BraA05g025100.3C	1.694	0.01770	Up	POP2
BraA09g039860.3C	0.566	0.0067	Down	GGAT

### The PPI network of succinylated proteins in response to OBZ stress

**Figure 5:**
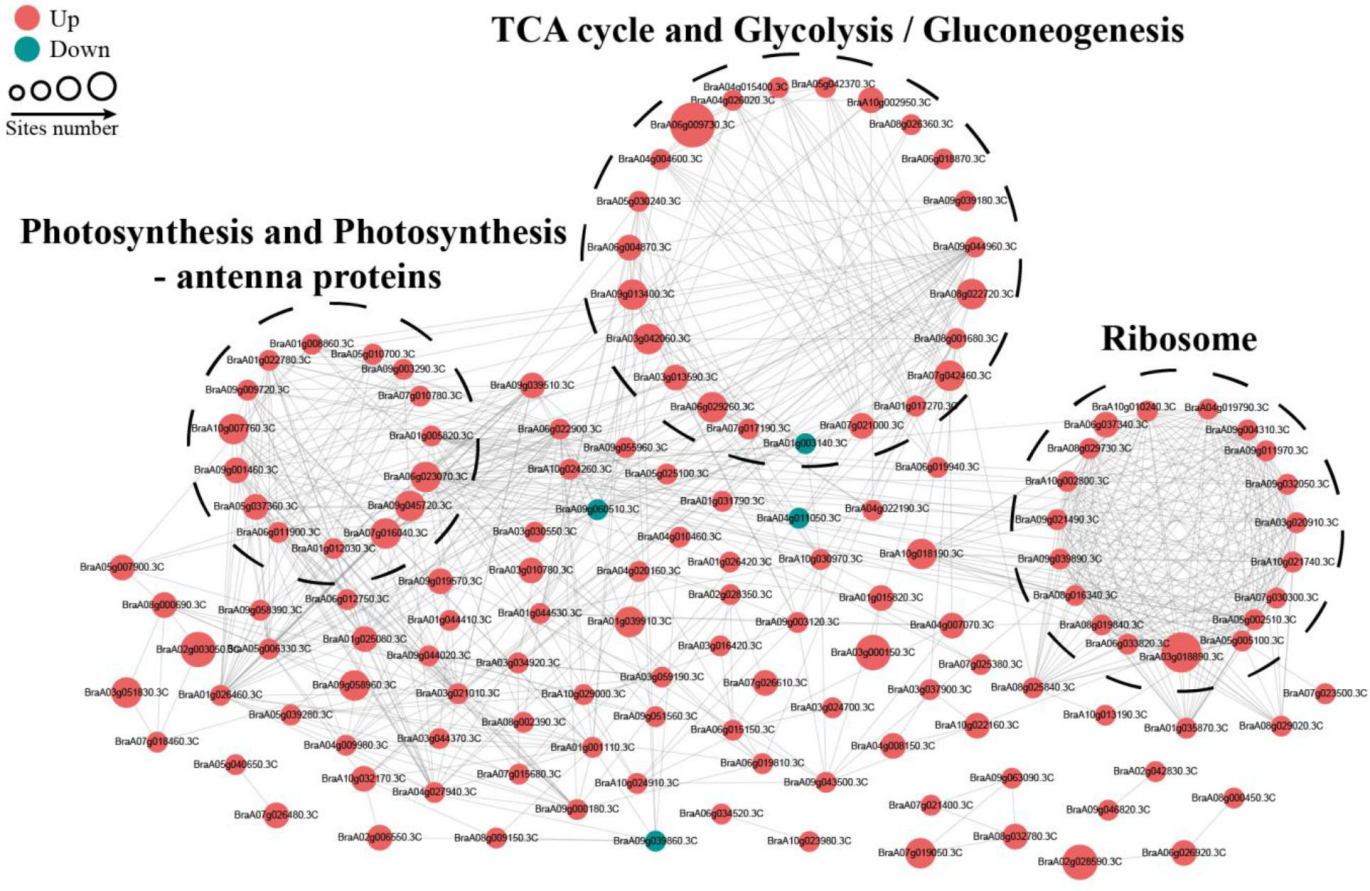
PPI network of succinylated proteins in response to OBZ stress.

To reveal the relationship between DSPs involved in the same biological process, we assembled a PPI network for all identified differentially modified proteins based on the STRING database and visualized them by the R package “networkD3”. Most DSPs were upregulated under OBZ stress. Three highly connected subnetworks, including photosynthesis and photosynthesis-antenna proteins, glycolysis/gluconeogenesis and tricarboxylic acid (TCA) cycle, and ribosomes, are displayed (**Figure 5**).

## Discussion

Plants are well known to produce energy (ATP) through photosynthesis and respiration for normal life activities, which were shown to be the two main inhibition sites of OBZ in our previous study (Zhong et al., 2019). The results of this study also showed that the main differentially modified proteins were distributed in the chloroplast, mitochondria, and cytoplasmic matrix (**Figure 2E**). The light-harvesting proteins LHCB1, LHCB2, LHCB3, PSII subunits psb28, psbQ, psbO, psbS, PSI subunits psaD are involved in the progression of photosynthetic carbon fixation, and many ATP synthase subunits are involved in energy generation (**Figure 4B** and **Supplementary Table 4**). All showed significant Ksuc modifications under OBZ treatment. Intriguingly, none of these were detected with changes in previous proteomic analysis (Lozano et al., 2021), which means that, compared with the single detection of protein expression, PTMs represented by Ksuc play an essential role in regulating metabolic pathways in plants under stress. PTMs can regulate the activity, localization, and folding of proteins and can also affect the interaction between proteins and other biological macromolecules. Most PTMs are under the control of variable environmental and stress conditions (Zhou et al., 2018). Previously, by measuring the changes in mitochondrial Ksuc in mice under starvation and nonstarvation conditions, researchers found that the Ksuc degree in the liver and kidney was significantly increased under starvation conditions (Park et al., 2013), suggesting that Ksuc may be responsible for responding to cellular energy status (Zhang et al., 2011; Weinert et al., 2013) and involved in the regulation of various metabolic enzyme activities in cells (Sadhukhan et al., 2016), which was supported by the results of this experiment.

In addition, OBZ has been found to directly alter the energy production processes in plants (Zhong et al., 2019), which further leads to obvious effects on important metabolic processes such as carbon and nitrogen metabolism that depend on the currency of energy circulation. Our results showed that OBZ significantly altered the Ksuc levels of proteins involved in carbon metabolism pathways (**Figure 4C** and **Supplementary Table 4**). Hypersuccinylation occurred in glyceraldehyde 3-phosphate dehydrogenase (GAPDH), enolase (ENO), and pyruvate dehydrogenase complex E1 component alpha subunit (PDHA) under OBZ treatment. Previous studies have found that the upregulated levels of Ksuc at the sites in 1K80, K81, and K335 of ENO could significantly inhibit its activity (Kurmi et al., 2018), and the increased Ksuc level of the PDHA complex would also enhance its activity, thus promoting the conversion of pyruvate and lactate into acetyl-CoA, which enters the TCA cycle for aerobic oxidation (Park et al., 2013). As an important link in the aerobic oxidation of glucose, the TCA cycle can produce a large amount of reduced nicotinamide adenine dinucleotide (NADH) and reduced flavin adenine dinucleotide (FADH_2_), which provides electrons for the respiratory chain and promotes oxidative phosphorylation to synthesize ATP. In our results, OBZ promoted the Ksuc levels of ACO, oxoglutarate dehydrogenase (OGDH) E1k, OGDH E2k, LSC2, DLD, SDHA/SDH1, MDH1, and MDH2 in the TCA cycle (**Figure 4C** and **Supplementary Table 4**); in contrast, the Ksuc level of IDH3 was decreased. Previous studies have shown that the hypersuccinylation of IDH1, IDH2, OGDH, SDHA, SDHB, and MDH2 attenuates their catalytic activity, leading to impaired mitochondrial respiration (Zhou et al., 2016; Wang et al., 2019; Ou et al., 2020). Notably, OGDH consists of three subunits (E1k, E2k, and E3), of which the E1k subunit catalyses the rate-limiting step in the TCA cycle (i.e., OGDH is a rate-limiting enzyme), which largely determines the carbon flux rate throughout the TCA cycle. Thus, the significant Ksuc and catalytic inhibition of these enzymes would all lead to the attenuation of the TCA cycle and the inhibition of mitochondrial function (Ou et al., 2020). Meanwhile, research on cucumber and tobacco also showed that OBZ suppresses the respiration rate (Zhong et al., 2019; Zhong et al., 2020; Wang et al., 2022). Intriguingly, some NADPH-producing enzymes, such as 6-phosphogluconate dehydrogenase (6-PGD), were also modified by Ksuc under OBZ stress (**Supplementary Table 2**). As the rate-limiting step of dehydrogenation, 6-PGD can generate 1 molecule of NADPH in the pathway of the pentose phosphate (PPP), which is a strong reducing agent to maintain the redox state of cells. The significant Ksuc in the 6-PGD means that Ksuc may also be involved in the control of the antioxidant system by regulating the metabolic enzymes that produce NADPH. Moreover, our results also showed that OBZ upregulated the Ksuc level of ribulose-phosphate 3-epimerase (RPE) in the PPP pathway (**Supplementary Table 2**). RPE can convert 5-phospho-ribulose (5-P-Ru) to 5-phospho-ribosyl (5-P-R), which is used for synthesizing purine and pyrimidine nucleotides. Therefore, in this experiment, OBZ may lead to the redistribution of intracellular carbon flow in pakchoi, and Ksuc plays an important role as a regulator.

In addition to C metabolism, amino acids are also essential to plants; for example, some of them can act as intermediates for the end products of certain metabolic pathways or participate in the regulation of various metabolic and other physiological and biochemical processes (Amir et al., 2018). The biosynthesis of amino acids is usually limited by energy availability (Araus et al., 2020), and the lack of energy caused by OBZ would undoubtedly inhibit this progress. Organisms usually adjust amino acid metabolism to address abiotic stress challenges, which is bound to lead to changes in its composition and the flux of the free amino acid pool (Heinemann and Hildebrandt, 2021). OBZ induces hypersuccinylation of most amino acid synthesis- and transamination-related proteins, for example, the serine hydroxymethyl transferase glyA/SHMT (BraA01g001110.3C, with FC = 1.757) (**Figure 4C** and **Supplementary Table 5**). The desuccinylation in the K280 site of SHMT2 will upregulate its activities (Yang et al., 2017), suggesting that Ksuc is also crucial in amino acid synthesis/metabolism.

Under environmental stress, organisms usually accumulate a large amount of ROS by performing complex physiological and biochemical processes. Several experiments have shown that OBZ increases the level of oxidative stress in organisms (Zhong et al., 2020; Wang et al., 2022). To counteract oxidative damage caused by ROS, organisms have evolved a series of complex defence mechanisms, including superoxide dismutase (SOD), catalase (CAT), peroxidase (POD), and the enzymes associated with the ascorbate-glutathione (ASA-GSH) cycle. Among them, SOD, as the first line of defence against ROS, consists of multiple homologous genes, all of which are regulated by Ksuc. In this experiment, we observed hypersuccinylation of SOD1 and SOD2 under OBZ treatment (**Figure 4A** and **Supplementary Table 3**). Desuccinylation on the K123 site of SOD1 can activate its activity to promote ROS scavenging function (Lin et al., 2013). Furthermore, OBZ induced hypersuccinylation of glutathione peroxidase (GPX) and glutathione sulfur transferase (GST) (**Figure 4A** and **Supplementary Table 3**). In addition, we measured the MDA and ROS (${\text{O}}_{2}^{-}$, H_2_O_2_) content and the activities of antioxidant enzymes (SOD, POD, CAT) under both control and OBZ treatment. The results showed that OBZ treatment significantly increased the MDA and ROS (${\text{O}}_{2}^{-}$, H_2_O_2_) content (**Supplementary Figures 1A, B, C**), and the activities of POD and CAT were upregulated under OBZ stress (**Supplementary Figures 1E, F**). Besides that, it has been found that although there was no significant difference in the protein expression level of the CAT under OBZ treatment (unpublished data), the hypersuccinylation that occurred on the CAT protein still promoted the improvement of enzyme activity, all of these results implied that succinylation plays an essential role in the regulation of plant antioxidant defense. Moreover, the heat shock protein HSPA1S was hypersulfinylated at multiple sites under OBZ treatment (**Supplementary Table 3**). Previous studies have shown that OBZ can cause increased transcription levels of HSP70 and some other small-molecule heat shock protein (sHsp) genes *in vivo* (Martín-Folgar et al., 2018). Therefore, the multisite Ksuc reaction of HSPA1S may play a key auxiliary role in the process of plants coping with OBZ stress.

The nucleosome, as an important carrier of genetic material, is the basic structural unit of eukaryotic chromatin, which is composed of DNA-wrapped histone octamers. The structure of nucleosomes dynamically disassembles and assembles to regulate DNA-related biological processes, such as gene expression, DNA replication, and DNA damage repair (Zentner and Henikoff, 2013). Previous studies have shown that the PTMs of histones are important for regulating the dynamic structural changes of nucleosomes, which can affect the interactions between histones, the interactions between DNA and histones, and the structure formation of the higher order of chromatin (Bowman and Poirier, 2015). Our experiment showed that a total of 4 histone domain-containing proteins and histones (fragments) were significantly modified by Ksuc under OBZ treatment (**Supplementary Table 2**), which included GTP-binding protein SAR1 (BraA08g032780.3C), histone H2B (BraA07g019050.3C, BraA07g021400.3C) and H4 (fragment) (BraA09g063090.3C). Jing et al. (2020) studied the effect of Ksuc of K77 site on the structural stability of nucleosomes by fluorescence resonance energy transfer (FRET). It was found that compared with the nucleosome composed of histones without modification, the Ksuc at K77 site significantly reduced the stability of the nucleosome structure, and made the nucleosome easy to depolymerize at a lower salt concentration. In addition, they also found that the Ksuc of H2B protein at position 34 (H2B K34suc) could affect the interaction between histones and DNA, thus affecting the stability of nucleosome structure (Jing et al., 2018). All the results implied that OBZ can participate in the regulation of gene expression by regulating the Ksuc level of corresponding histones.

**Figure 6:**
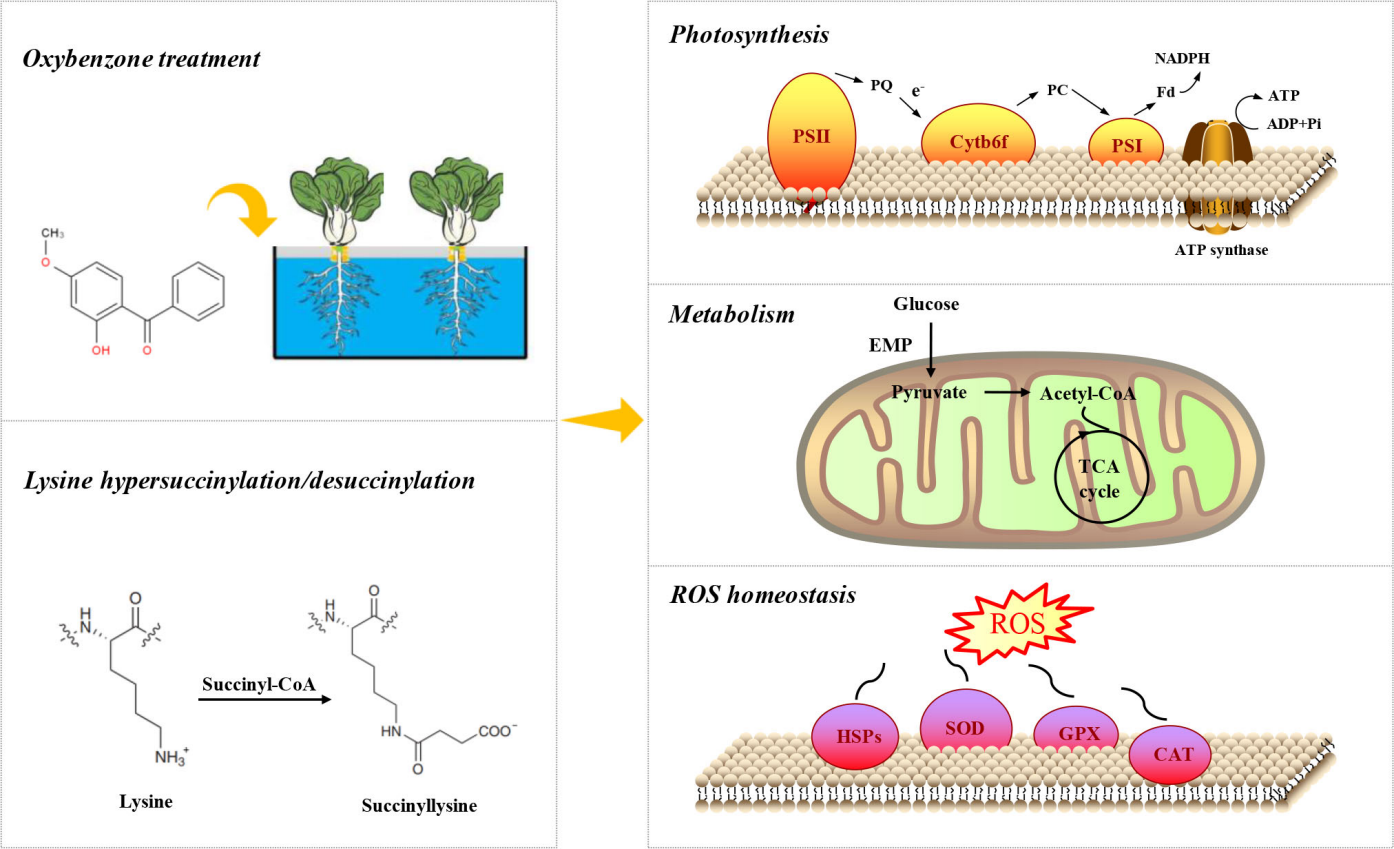
Simplified map in pakchoi response to OBZ stress.

Based on the above results, we speculated that Ksuc may contribute to the integration of environmental signals and globally control the activities of metabolism-related proteins, thereby coordinating the metabolic responses of plants to different kinds of stress (**Figure 6**). However, although some enzyme activities and metabolic pathways can be regulated by Ksuc (Yang and Gibson, 2019), the presence of Ksuc does not mean absolute changes in enzymatic activity. For example, a previous study proved that Ksuc on the sites of SIRT5 KO-induced citrate synthase or ATP synthase does not alter the activity of these target enzymes (Sadhukhan et al., 2016), and the loss of proteome annotation and undefined functional Ksuc can both affect the final regulatory metabolism network. In short, our study provided a dataset of Ksuc changes in higher plants under OBZ stress, demonstrating the flexible regulation of PTMs in the process of plant adaptation to stress and providing insights for future research on the function of individual proteins.

## Data availability statement

The original contributions presented in the study are publicly available. This data can be found here: http://proteomecentral.proteomexchange.org PXD036378.

## Author contributions

XZ and FZ designed the research plan, and modified the manuscript. SL and YZ performed most of the experiments. SL and XZ analysed the data, and wrote the article. YX, SR, MH and QL performed some of the experiments. All authors contributed to the article and approved the submitted version.
