# Supplementary material for: The analysis of lysine succinylation modification reveals the mechanism of oxybenzone damaging of pakchoi (Brassica rapa L. ssp. chinensis)
Source: Front Plant Sci. 2022 Dec 8;13:1001935. doi: 10.3389/fpls.2022.1001935 (PMC9772522; doi:10.3389/fpls.2022.1001935)
Supplement: Supplementary file 2 [file DataSheet_2.docx]

Supporting Information for

**The analysis of lysine succinylation modification reveals the mechanism of oxybenzone damaging of pakchoi (*Brassica rapa* L. ssp. *chinensis*)**

Shuhao Li, Yuqi Zhou, Yang Xu, Shengxiang Ran, Maomao Hou, Qingming Li, Xin Zhong and Fenglin Zhong

Corresponding author: Xin Zhong and Fenglin Zhong.

Email: [751538948@qq.com](mailto:751538948@qq.com) (X. Zhong); [zhong591@fafu.edu.cn](mailto:zhong591@fafu.edu.cn) (F.L. Zhong).

**This file includes:**

Supplementary Fig. 1.

Supplementary methods (Measurement of the content of MDA, H_2_O_2_, O_2_^−^, and the activity of SOD, POD, CAT).

Supplementary Table 1 to 5 (excel files).

**Supplementary Fig. 1** The MDA content (A), O_2_^−^ production rate (B), H_2_O_2_ content (C), SOD activity (D), POD activity (E), and CAT activity (F) on both control and OBZ treatment. Values are the mean ± SD (n = 3). Different letters indicate significant differences at *P* < 0.05 according to Tukey's test.

**Measurement of the content of MDA, H_2_O_2_, O_2_^−^, and the activity of SOD, POD, CAT**

The contents of malondialdehyde (MDA) and hydrogen peroxide (H_2_O_2_), the production rates of superoxide anion radical (O_2_^−^), and the activities of superoxide dismutase (SOD), peroxidase (POD), and catalase (CAT) were measured using respective reagent kits (Comin Biotechnology Co. Ltd, Suzhou, China) following the manufacturer’s instructions.
